# Adenylosuccinate lyase deficiency affects neurobehavior via perturbations to tyramine signaling in *Caenorhabditis elegans*

**DOI:** 10.1371/journal.pgen.1010974

**Published:** 2023-09-29

**Authors:** Corinna A. Moro, Sabrina A. Sony, Latisha P. Franklin, Shirley Dong, Mia M. Peifer, Kathryn E. Wittig, Wendy Hanna-Rose

**Affiliations:** Department of Biochemistry and Molecular Biology, The Pennsylvania State University, University Park, Pennsylvania, United States of America; Brown University, UNITED STATES

## Abstract

Adenylosuccinate lyase deficiency is an ultrarare congenital metabolic disorder associated with muscle weakness and neurobehavioral dysfunction. Adenylosuccinate lyase is required for *de novo* purine biosynthesis, acting twice in the pathway at non-sequential steps. Genetic models can contribute to our understanding of the etiology of disease phenotypes and pave the way for development of therapeutic treatments. Here, we establish the first model to specifically study neurobehavioral aspects of adenylosuccinate lyase deficiency. We show that reduction of *adsl-1* function in *C*. *elegans* is associated with a novel learning phenotype in a gustatory plasticity assay. The animals maintain capacity for gustatory plasticity, evidenced by a change in their behavior in response to cue pairing. However, their behavioral output is distinct from that of control animals. We link substrate accumulation that occurs upon *adsl-1* deficiency to an unexpected perturbation in tyrosine metabolism and show that a lack of tyramine mediates the behavioral changes through action on the metabotropic TYRA-2 tyramine receptor. Our studies reveal a potential for wider metabolic perturbations, beyond biosynthesis of purines, to impact behavior under conditions of adenylosuccinate lyase deficiency.

## Author summary

Adenylosuccinate lyase deficiency is an ultra-rare metabolic disorder associated with muscle and neural dysfunction, including muscle weakness, psychomotor delay, and repetitive vocalizations and behaviors. The disorder has been modeled successfully in the nematode *C*. *elegans* in terms of recapitulating the metabolic signatures and the muscular deficits. However, a model for studying neurobehavioral aspects of the disorder has not been established. We have used *C*. *elegans* to explore the cause of neurobehavioral phenotypes associated with adenylosuccinate lyase deficiency. We found that reduction of *adsl-1* function in *C*. *elegans* results in an exaggerated response in a gustatory plasticity assay. Naïve control animals are attracted to salt but become indifferent to salt after being starved in the presence of salt. When *adsl-1* function is compromised, animals change behavior after starvation-cue pairing but show a repulsive response in contrast to the indifferent response of control animals. The *adsl-1* animals are capable of learning from experience but their response to that learning is distinct. We also discovered that this abnormal behavior is caused by a deficiency in production of the metabolite tyramine and is shared by other mutants with tyramine signaling deficits. Finally, we demonstrate that it is likely the accumulation of a toxic substrate that causes the phenotype as opposed to perturbation of purine metabolism. Development of this model and these findings have exciting implications for therapeutic development for adenylosuccinate lyase deficiency.

## Introduction

Congenital metabolic disorders (CMDs) arise from genetic mutations that result in reduction or absence of enzymatic activity, leading to phenotypic clinical manifestations. Although most CMDs are rare individually, they are collectively common and affect 1 in 1500 births [1]. CMDs are often underdiagnosed due to the lack of metabolic testing required for diagnosis as well as the overlap of clinical manifestations with other childhood disorders leading to misdiagnosis. CMDs are frequently named after the pathways that are perturbed by loss of enzymatic activity. However, perturbation of one metabolic pathway can have implications in other, independent pathways and can lead to a global metabolic dysfunction. Our study focuses on an ultra-rare CMD in purine metabolism, adenylosuccinate lyase deficiency, that is associated with both neural and muscular deficits, leading to adverse health effects.

Adenylosuccinate lyase deficiency (ASLD; OMIM #103050) is an autosomal recessive disorder that was first described in 1984 by Jaak Jaeken and Georges Van Den Berghe in three children, two who were related, with severe psychomotor delays and autism [2]. The most common clinical manifestations of ASLD are hypotonia and intellectual disability. Other symptoms can include ataxia, autistic-like behavior, temper tantrums, cerebellar hypotrophy, muscle wasting, growth failure, encephalopathy, seizures, and auto-aggressivity [2–5]. Approximately half of ASLD patients suffer from intractable seizures [6]. Controlling the frequency and severity of the seizures is often the only treatment available.

Adenylosuccinate lyase (ADSL; EC 4.3.2.2) catalyzes two non-sequential steps in the *de novo* purine synthesis pathway (DNPS): it converts succinylaminoimadazole carboxamide ribotide (SAICAR) into aminoimidazole carboxamide ribotide (AICAR) and adenylosuccinate (S-AMP) into adenosine monophosphate (AMP). A decrease in enzymatic activity of ADSL in humans leads to build up of dephosphorylated forms of the succinylpurine substrates: succinylaminoimidazole carboxamide riboside (SAICAr) and succinyladenosine (S-Ado) [7].

ADSL, and DNPS more generally, are highly conserved throughout the tree of life, and models to study ADSL deficiency have been established in a few systems [8–12]. Dutto *et al*. revealed that ciliogenesis is impaired in the absence of ADSL, and knocking down *adsl* expression in zebrafish led to phenotypes associated with defective ciliogenesis, including inverted liver placement and inverse heart loops [10]. Neurodevelopmental effects of ADSL are documented in zebrafish and in chick embryos [10]. In *C*. *elegans*, *adsl-1* deficiency results in developmental, reproductive and mobility phenotypes [9,12].

Two main hypotheses have been put forward to explain the effects of ADSL deficiency; either disruptions in purine homeostasis or accumulation of toxic intermediates could explain disease etiology. Studies in cultured cells recapitulate accumulation of substrates seen in patients in some cases, but not in others [10,11]. The lethality of deletion of the gene encoding adenylosuccinate lyase in yeast is suppressed by deletion of upstream genes in the pathway, supporting the SAICAR toxicity hypothesis [8]. By using supplementation experiments and inhibitors to block DNPS upstream of ADSL activity, Dutto et al attributed neurogenesis defects to the likely buildup of SAICAr [10]. In *C*. *elegans*, different phenotypes have different etiologies. The reproductive phenotypes have been attributed to perturbations in purine homeostasis and the mobility phenotypes to toxic accumulation of SAICAR/r [10,12]. Interestingly, steady state purine levels are not generally perturbed in patients or in the *C*. *elegans* model [9,12–14]. There are still significant gaps in our knowledge related to the etiology behind the neurobehavioral manifestations of ASLD as well as other CMDs of purine metabolism.

We, and others, have shown that knockout of *adsl-1*, which encodes the *C*. *elegans* adenylosuccinate lyase, results in severe mobility defects [9,12], precluding analysis of some behavioral phenotypes. However, a reduction of function model could ameliorate this drawback and better reflect the status of residual gene function in ASLD. In fact, when *adsl-1* function is reduced, as opposed to removed, levels of both *adsl-1* substrates are increased but mobility defects are less severe [9,12]. Here we develop this model further to study neurobehavioral outcomes related to neural plasticity upon reduction of ADSL function. And we extend the findings to loss of function mutants, formally implicating both *adsl-1* and the tyrosine-derived neurotransmitter tyramine in behavioral plasticity in *C*. *elegans*.

A number of behavioral paradigms have been developed in *C*. *elegans* to study learning [15,16]. Naïve control animals are attracted to low levels (less than 200 mM) of sodium chloride [17], and this behavior is modulated based on life experience. For example, this unconditioned response is altered by brief (minutes) as well as extended (hours) periods of starvation resulting in stronger attraction to salt. In contrast, if the period of starvation is paired with exposure to sodium chloride, the animals display an altered conditioned response of avoidance or indifference to salt [16,18,19]. Paradigms involving long periods of training between unconditioned and conditioned stimuli are often referred to as associative learning. In contrast paradigms involving short periods of training are referred to as gustatory plasticity, a specialized category of salt aversion associative learning [16,18,19]. We assess sensory and behavioral phenotypes associated with reduction of ADSL-1 function in *C*. *elegans* and establish a novel response in a gustatory plasticity paradigm. We further show that a deficit in activity of a tyrosine-derived biogenic amine underlies neurobehavioral phenotypes in this *C*. *elegans* purine CMD model.

## Results

### *adsl-1(RNAi)* animals can sense and respond to salt

**Fig 1:**
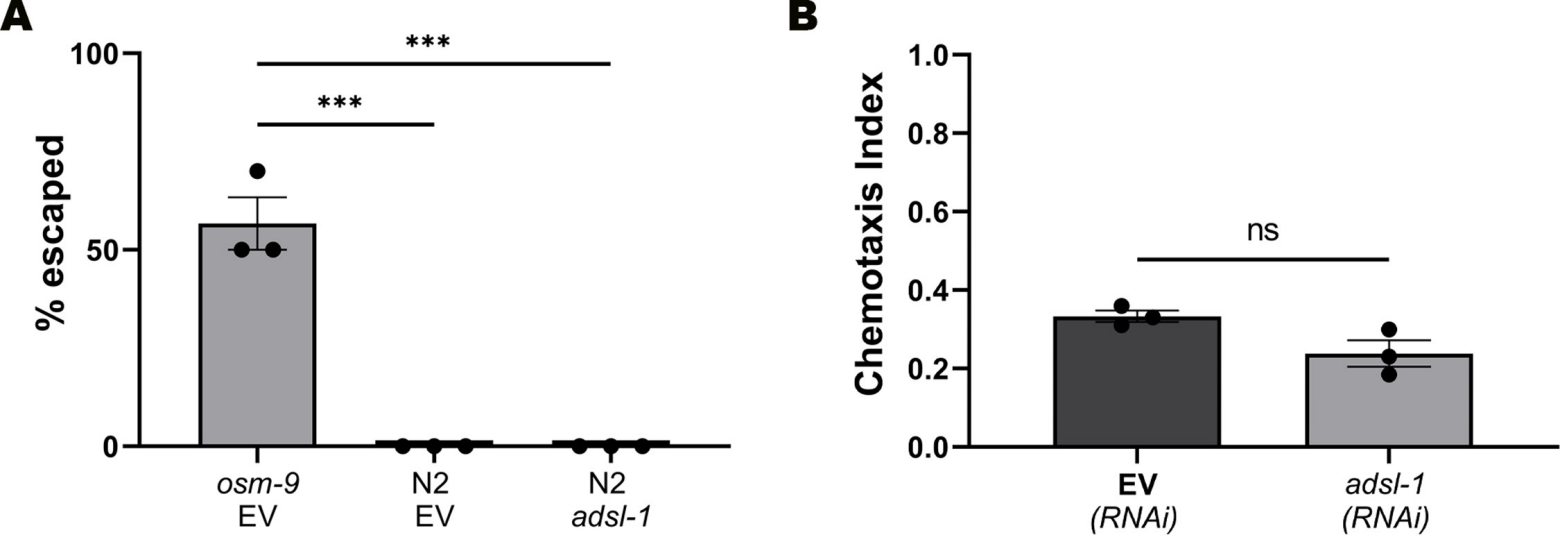
*Adsl-1(RNAi)* animals can sense and respond to salt. **(A)** *osm-9* mutants and N2 animals exposed to empty vector (EV) control RNAi or *adsl-1* RNAi were tested for osmosensation using an osmotic ring test with 4 M NaCl. Average percentage of animals that crossed the high osmolarity ring (escaped) is plotted. Each dot represents the value from a single biological replicate with 10 animals per condition. **(B)** EV control and *adsl-1* RNAi were examined in a chemotaxis assay for attraction to 100 mM NaCl. Each dot represents the value from a single biological replicate with >120 animals per condition per replicate. Error bars represent standard error of mean (SEM). ns, nonsignificant, *** p<0.001 using one-way ANOVA with Tukey’s Test (A) or unpaired t-test (B).

Because *adsl-1*(RNAi) animals have locomotion deficits [9,12] we first evaluated the *adsl-1(RNAi)* model for suitability in movement-based behavioral assays that require intact sensory functions. *C*. *elegans* will avoid osmotic changes [20], and normal animals avoid sodium chloride concentrations above 200 mM [19]. We performed an osmotic ring test using 4 M sodium chloride to test the ability of control and *adsl-1*(RNAi) animals to sense the osmolarity and remain in the low osmolarity environment. We also included osmotic avoidance-defective *osm-9(ky10)* animals as a control [21]. *osm-9(ky10)* frequently cross the 4 M NaCl ring (Fig 1A). However, the response of *adsl-1(RNAi)* animals was indistinguishable from the control animals indicating that they can sense high sodium chloride as a noxious stimulus and will remain in areas of low osmolarity (Fig 1A).

We next examined response to sodium chloride upon reduction of *adsl-1* using a chemotaxis assay. We starved *adsl-1(RNAi)* hermaphrodites in chemotaxis (CTX) buffer for 15 minutes, then placed them into the central arena on chemotaxis plates with two control quadrants prepared with water and two test quadrants prepared with 100 mM NaCl. We calculated the chemotaxis index at 30 minutes. Although *adsl-1(RNAi)* animals have impaired mobility, 30 minutes was adequate time for a chemotactic response, and the *adsl-1(RNAi)* and control animals displayed indistinguishable positive chemotactic indices (Fig 1B). We also examined mechanosensory function in nose touch and posterior touch assays that require movement in response and found no defects (S1 Fig). Overall, we conclude that *adsl-1(RNAi)* animals do not have detectable gross sensory defects and the *adsl-1(RNAi)* mobility defects do not prevent assessment of chemosensory activity.

### Gustatory plasticity is altered upon reduction of *adsl-1* activity

**Fig 2:**
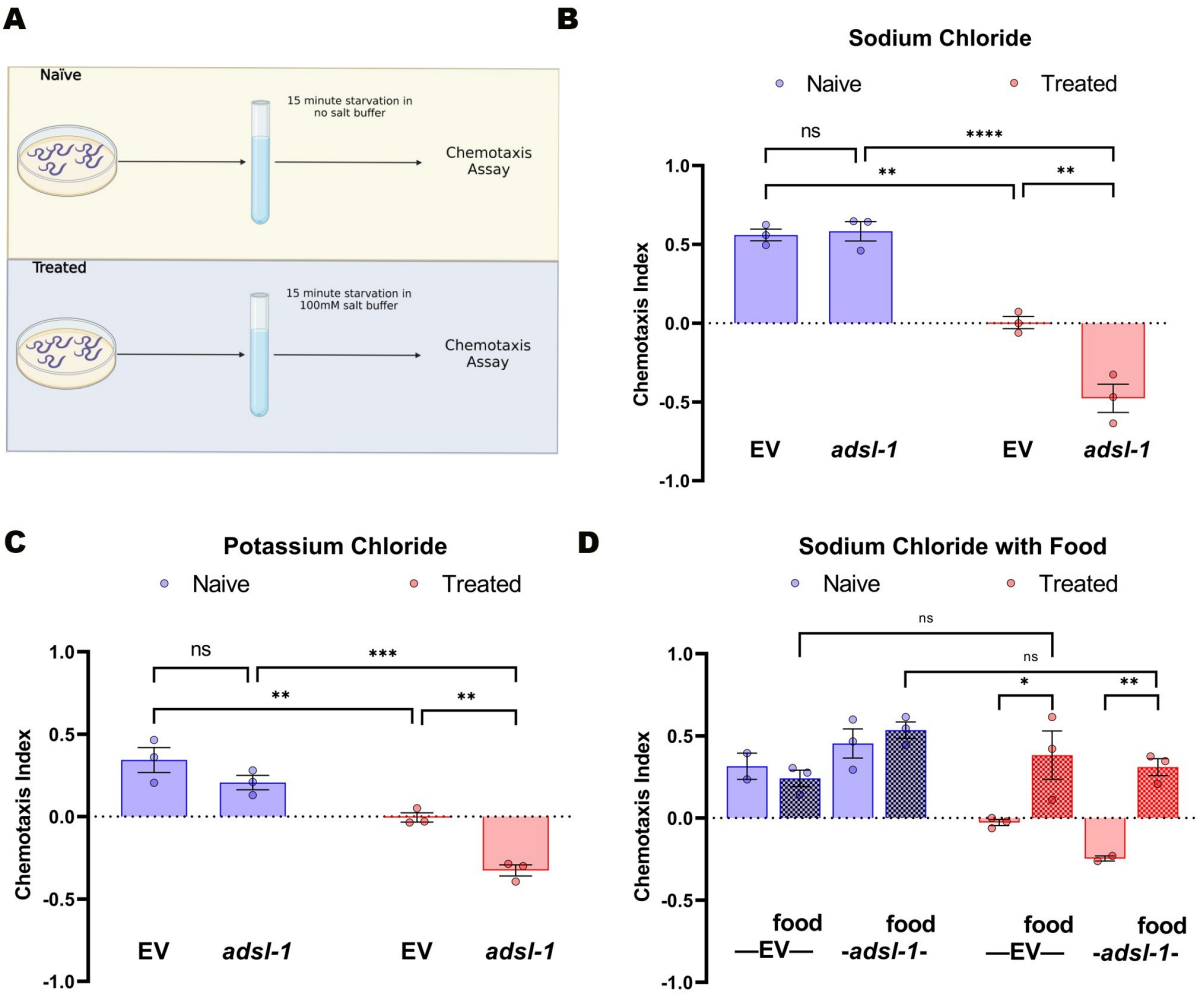
*Adsl-1(RNAi)* animal response in a gustatory plasticity assay is robust but distinct from control animals. **(A)** Diagram of the gustatory plasticity assay (created with Biorender.com). Animals are washed off the plates with either CTX buffer or CTX buffer with 100 mM salt and left in a conical tube for 15 minutes with or without food. They are then placed on a chemotaxis assay plate for 30 minutes. **(B)** Gustatory plasticity assay with 100 mM sodium chloride as the conditioned stimulus. *adsl-1*(RNAi) animals respond robustly but distinctly from control animals. **(C)** Gustatory plasticity assay with 100 mM potassium chloride as the conditioned stimulus. *adsl-1*(RNAi) animals respond robustly but distinctly from control animals. **(D)** Gustatory plasticity assays in the absence or presence (hatched bars) of food. Neither *adsl-1*(RNAi) nor control animals alter their chemotactic response when food is included in the preincubation with salt. Each dot represents the value from a single biological replicate that included (B) 22<n<238, (C) 33<n<266, (D) 15<n<65 animals per condition per replicate. Error bars represent standard error of the mean (SEM) * p< 0.05, ** p<0.01, *** p < .0001, **** p<0.0001 using (B-C) 2-way or (D) 3-way ANOVA with Bonferroni correction.

*C*. *elegans* regulate their response to sensory stimuli based on previous experiences, as evidenced, for example, by a change in chemotactic response to sodium chloride after cue-pairing of low levels of sodium chloride and starvation [15,22,23]. To further probe *adsl-1* animals for neuro-behavioral phenotypes, we examined their responses in a gustatory plasticity assay (Fig 2A). As expected, we found that the chemotactic response of control animals to 100 mM NaCl is positive when animals are starved or when they are provided with food during the 15-minute training period (Fig 2B and 2D). However, if 100 mM sodium chloride is added into the buffer during the training, the starved animals subsequently display a suppressed chemotaxis index (<10% of magnitude of normal). The fed animals maintain the positive chemotaxis index (Fig 2B and 2D). Thus, pairing of the conditioned and unconditioned stimulus, starvation and salt, alters the behavioral output relative to the naïve animals, causing them to become indifferent to sodium chloride in the chemotaxis assay.

*adsl-1(RNAi)* animals had a distinct and surprising response in this gustatory plasticity assay. Like the control animals, the chemotactic response of the *adsl-1(RNAi)* animals to 100 mM NaCl is positive when animals are starved or when they are provided with food during the 15-minute incubation (Fig 2B and 2D). As expected, if 100 mM sodium chloride is added to the buffer during training, only the starved animals subsequently display an altered chemotactic response relative to naïve *adsl-1(RNAi)* animals, demonstrating gustatory plasticity. Strikingly, although the animals change behavior in response to the cue pairing, the outcome is distinct from that of control animals. Pre-exposure to sodium chloride in the presence of the negative cue (starvation) causes the *adsl-1(RNAi)* animals to robustly avoid the sodium chloride (Fig 2B); they display a chemotaxis index of similar magnitude (80% of magnitude) but of the opposite direction (repulsion) relative to the attractive response observed without conditioning. We repeated the gustatory plasticity assay using 100 mM KCl instead of NaCl and observed similar results (Fig 2C). Both the control and *adsl-1*(RNAi) animals demonstrate gustatory plasticity, but the treated response of the control EV animals results is a CI that is less than 10% of the magnitude of naïve animals, demonstrating indifference to the KCl. In contrast, the *adsl-1*(RNAi) treated animals continue to have a robust CI at 60% of the magnitude of untreated but again in the opposite direction, demonstrating avoidance of the KCl. Thus, the altered gustatory plasticity phenotype is not unique to sodium chloride. We conclude that reduction of *adsl-1* function does not prevent gustatory plasticity, but it substantially alters the behavioral output in response to cue pairing compared to control animals.

### Tyramine is necessary for normal gustatory plasticity

**Fig 3:**
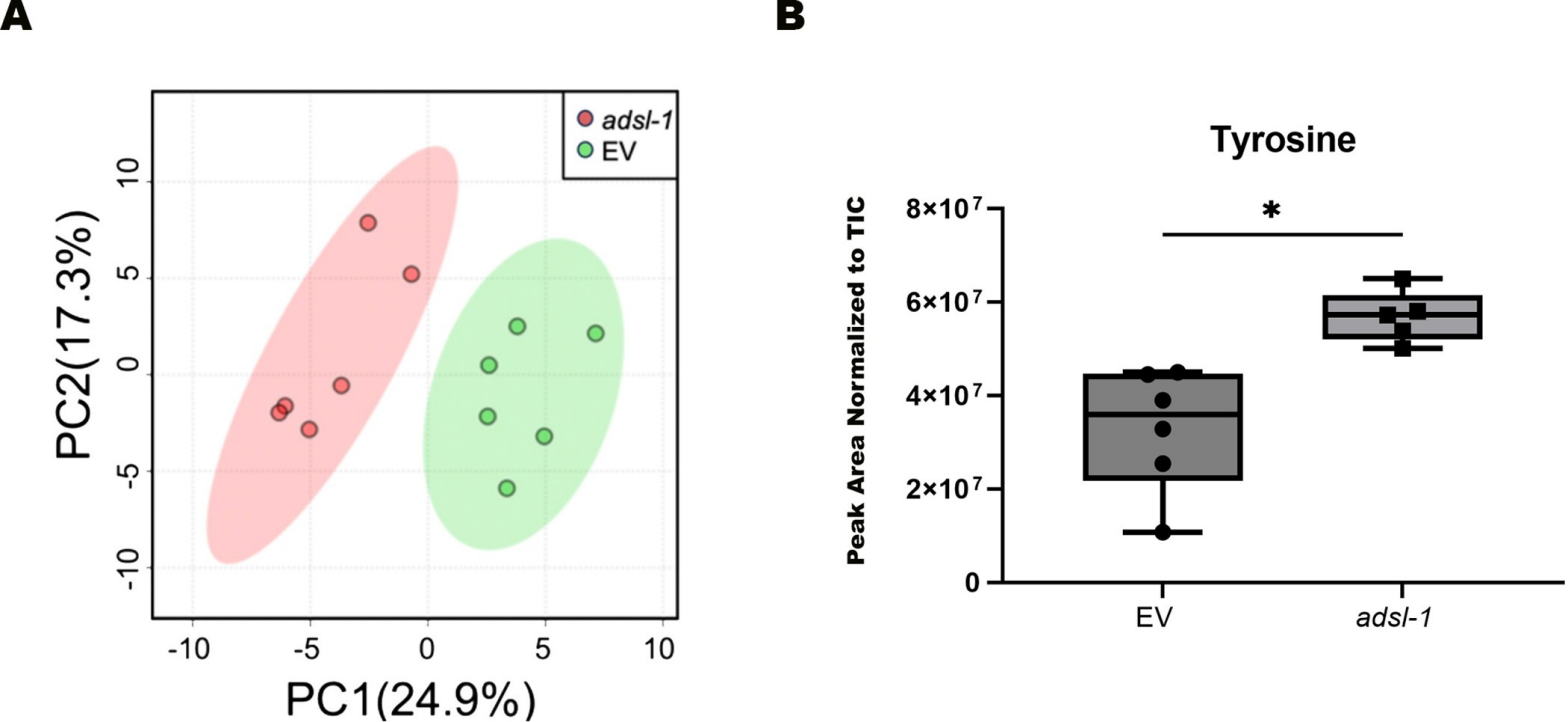
**(A)** Principal component (PC) analysis of untargeted LC-MS metabolomics data demonstrating that the *adsl-1*(RNAi) metabolome is distinct from EV control based on PC1. The variances explained by the indicated PC are shown in brackets. **(B)** Tyrosine levels increase in *adsl-1(RNAi)* animals compared to the empty vector (EV) control. Each dot represents peak are in one sample. Boxes show the upper and lower quartile values, lines indicate the median. Error bars indicate the maximum and minimum of the population distribution. *, 0.01<p<0.05, calculated using Welch’s t test.

We expect perturbation of purine biosynthesis to have widespread effects on metabolism. To characterize these changes and to generate hypotheses about the etiology of the behavioral phenotypes, we turned to metabolomics experiments using LC-MS. We previously revealed that increases in the ADSL substrates SAICAR and S-AMP are associated with reduction of *adsl-1* activity via comparisons of targeted metabolite levels [12]. Using principal component analysis of LC-MS data sets for *adsl-1*(RNAi) and EV control animals, we show here that *adsl-1*(RNAi) and EV control animals have metabolomes that can be distinguished from each other (Fig 3A). Components driving this difference include increases in the ADSL substrates SAICAR and S-AMP and a decrease in the product fumarate (Table 1), but other significant changes are also observed. One of the changes that caught our attention was a perturbation to tyrosine levels. Tyrosine is increased in *adsl-1(RNAi)* compared to control animals (Fig 3B). Tyrosine attracted our attention because the biogenic amines tyramine, octopamine and dopamine, which play roles in learning, memory and behavior in *C*. *elegans* [24–28], are derived from tyrosine. Intriguingly, *C*. *elegans* tyrosine decarboxylase mutants that are unable to produce either tyramine or octopamine from tyrosine display an altered response under a salt-aversive learning paradigm involving long training durations [29]. Moreover, one of the few tyramine-producing cells in *C*. *elegans*, the RIM neuron, is a key node in the neural circuit involved in associative learning [29]. These observations led us to investigate if biogenic amines play a role in the altered gustatory plasticity response observed upon reduction of *adsl-1* function.

**Table 1:** Metabolites with altered levels in *adsl*-*1*(RNAi) relative to control.

Metabolite^a^	T.stat	p.value
SAICAR^b^	17.962	6.1215e-09
Trehalose/ sucrose	4.797	0.000726
Riboflavin	-3.8885	0.003017
Pyroglutamic acid	-3.6808	0.004240
Xanthine	-3.6698	0.004318
Proline	-3.6271	0.004634
Acetylcarnitine	-3.2638	0.008519
Fumarate^c^	-3.0567	0.012113
GMP	2.9955	0.013446
Guanine	2.8285	0.017899
N-acetyl-L-ornithine	2.8120	0.018411
D-glyceraldehyde 3-phosphate	2.7332	0.021076
Glutamine	-2.6972	0.022421
Tyrosine	2.6680	0.023574
Lysine	2.6602	0.023891
Thiamine	-2.6469	0.024442
S-AMP^b^	2.5733	0.027735
Methylmalonic acid	-2.4324	0.035306
Uric acid	2.2905	0.044973
AMP^c^	-2.2836	0.045508
histidine	-2.2422	0.048820

^a^ Identified features with p < .05 based on Student’s T test in MetaboAnalyst (v4.0)

^b^ ADSL-1 substrate

^c^ ADSL-1 product

**Fig 4:**
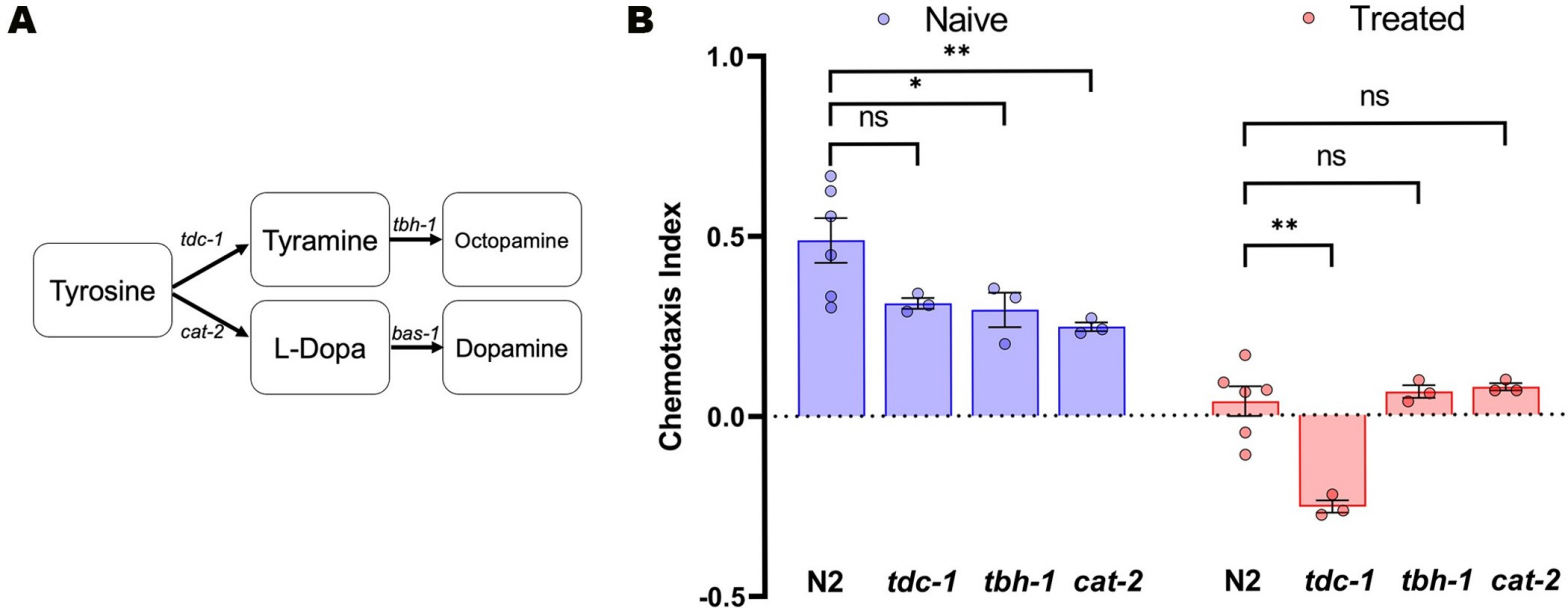
Tyramine is required for normal gustatory plasticity. (A) TDC-1 catalyzes the production of tyramine. TBH-1 converts tyramine to octopamine. Tyrosine can also be used to make the biogenic amine, dopamine. CAT-2 synthesizes levodopa (L-Dopa), and BAS-1 produces dopamine. Abbreviations: *tdc-1*, tyrosine decarboxylase-1; *tbh-1*, tyramine β-hydroxylase-1; *cat-2*, tyrosine hydroxylase; *bas-1*, aromatic amino acid decarboxylase. **(B)** Gustatory plasticity assay with mutants. *tdc-1* animals, which lack both tyramine and octopamine, have a robust but altered response in the gustatory plasticity assay. *tbh-1(n3247)* and *cat-2(n4547)* animals had gustatory plasticity responses comparable to the N2 control. Each dot represents the value from a single biological replicate with 20<n<386 animals per condition per replicate. Error bars represent standard error of mean (SEM). *, p<0.05, ** p<0.01 using two-way ANOVA with Bonferroni correction.

*tdc-1* encodes tyrosine decarboxylase in *C*. *elegans*, and *tdc-1*(*n3419)* mutant animals lack both octopamine and tyramine [27]. *tbh-1* encodes tyramine *β*-hydroxylase, and *tbh-1(n3247)* mutant animals specifically lack octopamine (Fig 4A) [27]. *cat-2* encodes tyrosine hydroxylase which produces the L-Dopa from tyrosine that is required to make dopamine (Fig 4A) [30]. We examined the behavior of mutations in the genes encoding these enzymes in our gustatory plasticity assay. The behavior of both *tbh-1(n3247)* and *cat-2(n4547)* animals was indistinguishable from controls, indicating normal chemotactic ability and gustatory plasticity (Fig 4B). *tdc-1(n3419)* animals had normal chemotactic activity. However, their response to the salt-starvation cue pairing was distinct (Fig 4B). The treated *tdc-1(n3419)* animals robustly avoid the sodium chloride; they display a chemotaxis index of similar magnitude (80%) but opposite direction relative to the response observed without conditioning. We conclude that tyramine, but not octopamine or L-Dopa, is required for normal gustatory plasticity, and in the absence of tyramine animals exposed to the cue pairing avoid salt as opposed to being indifferent.

**Fig 5:**
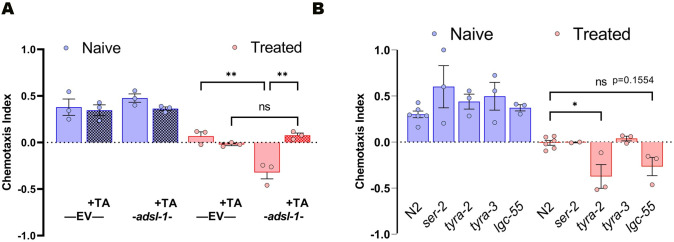
Tyramine supplementation reverts the *adsl-1*(RNAi) phenotype and *tyra-2* is necessary for gustatory plasticity. **(A)** Gustatory plasticity assay of empty vector (EV) control animals and *adsl-1(RNAi)* animals (solid bars) exposed to 10 mM tyramine (TA) (hatched bars) from the L1 stage. *adsl-1*(RNAi) animals treated with tyramine had a response comparable to control animals. Each dot represents the value from a single biological replicate with 18<n<307 animals per condition per replicate. **(B)** Gustatory plasticity assay of metabotropic receptor mutants *ser-2(pk1357)*, *tyra-2(tm1846)*, *tyra-3(ok325)* and ionotropic receptor mutant *lgc-55(n4331)*. *tyra-2(tm1846)* animals have a response distinct from that of control animals. Each dot represents the value from a single biological replicate with 15<n<238 animals per condition per replicate. Error bars represent standard error of mean (SEM). ns, nonsignificant * p<0.05, ** p<0.01, using three-way ANOVA with Tukey’s Test (A) and two-way ANOVA with Bonferroni correction (B).

To investigate the hypothesis that a tyramine deficiency causes the *adsl-1(RNAi)* phenotype, we cultured *adsl-1(RNAi)* animals with supplemental tyramine and examined their gustatory plasticity. After supplementation with tyramine, *adsl-1(RNAi)* animals behaved indistinguishably from the EV RNAi control (Fig 5A). We conclude that a lack of tyramine underlies the behavioral changes seen in *adsl-1(RNAi)*. To provide further evidence for the role of tyramine, we investigated the function of tyramine receptors in gustatory plasticity. There are four known tyramine receptors in *C*. *elegans*: metabotropic receptors SER-2, TYRA-2, TYRA-3, and ionotropic receptor LGC-55 [31]. We hypothesized that mutations in one or more receptors would have altered gustatory plasticity. The CI of the treated *ser-2(pk1357)* and *tyra-3(ok325)* mutants was suppressed to 10% or less than the magnitude of the naïve controls. Thus, these mutants were indiscernible from control N2 animals (Fig 5B). However, the CI for the treated *tyra-2(tm1846)* animals remains robust (at 85% of the magnitude of the control animals) but again in the opposite direction, demonstrating repulsed behavior after the starvation-salt cue pairing (Fig 5B). The CI for the *lgc-55*(*n4331)* mutant is intermediate and not statistically distinct from treated N2 animals. We conclude that *tyra-2* is required for normal gustatory plasticity, but we have not ruled out a role for *lgc-55*.

**Fig 6:**
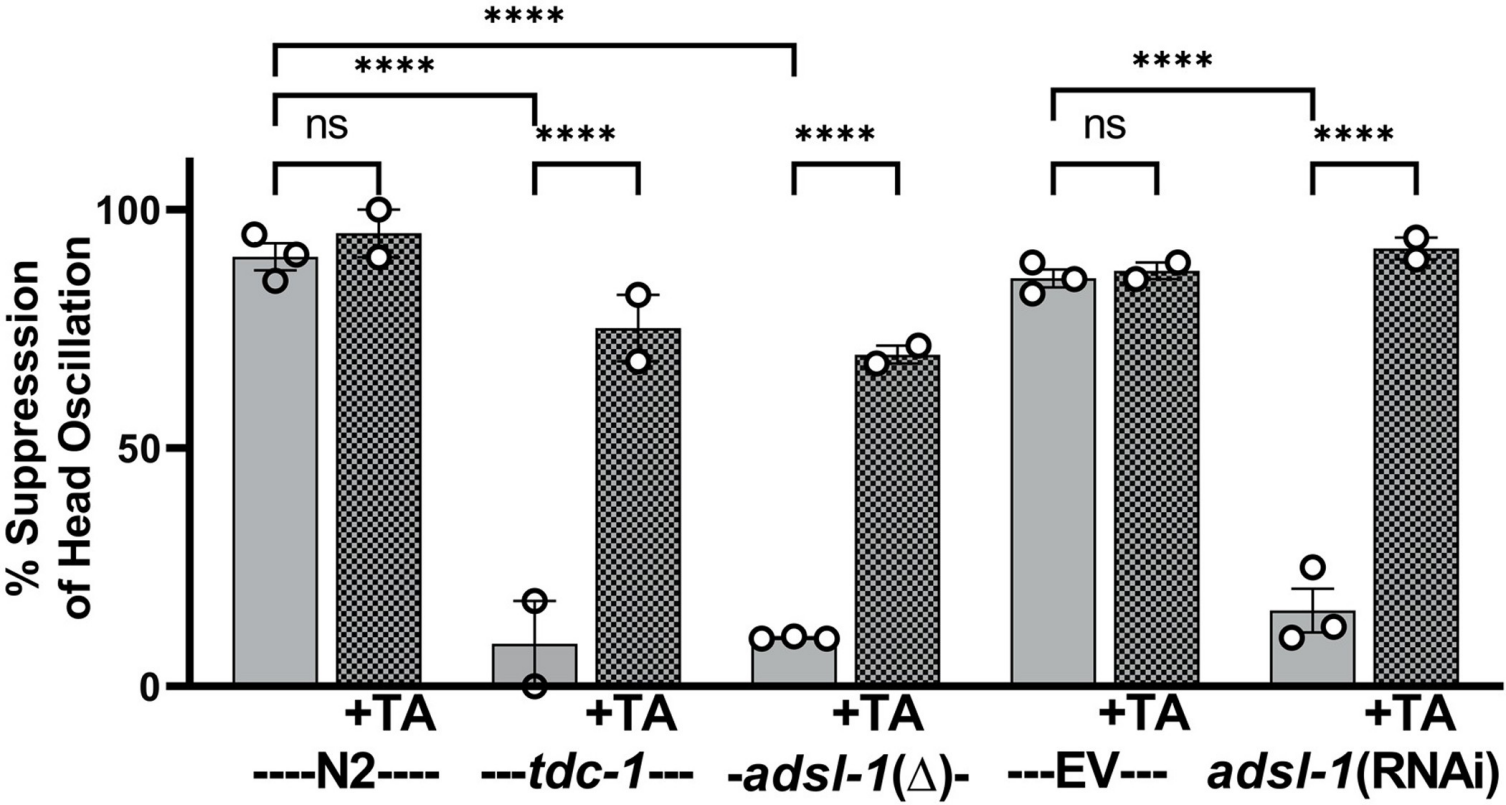
*adsl-1* mutant and *adsl-1*(RNAi) animals share a head suppression phenotype with *tdc-1* mutants, which lack tyramine. Percent of animals that suppress head oscillations when backing in response to a light touch, a “normal” response, is plotted. *adsl-1(tm3328)*, *adsl-1*(RNAi) and *tdc-1(n3418)* animals all fail to suppress head oscillations appropriately. +TA animals (hatched bars) were supplemented with 10 mM tyramine from the L1 stage. Each dot represents the value from a single biological replicate that included 20–40 animals per condition. Error bars represent standard error of the mean (SEM). **** p<0.0001, using two-way ANOVA with Bonferroni correction.

We have revealed a role for tyramine in gustatory plasticity and produced evidence in favor of the hypothesis that reduction in ADSL-1 activity results in a failure to produce sufficient levels of tyramine. If *adsl-1* mutants do not produce tyramine, they would be expected to share other phenotypes with *tdc-1* mutants. Wild-type animals oscillate their heads in foraging movements when moving forward but suppress these head oscillations when backing. Loss-of-function of *tdc-1* mutants are unable to suppress head oscillation during backing [27]. We tested *adsl-1(RNAi)* for head oscillation and found that they share this phenotype with *tdc-1(n3419)* (Fig 6). *adsl-1(tm3328)* loss-of-function mutants also fail to suppress head oscillations (Fig 6). We also tested the effect of tyramine in this assay and found that suppression of head oscillation is restored to *adsl-1(tm3328)*, *adsl-1*(RNAi), and *tdc-1(n3418)* animals when supplemented with tyramine (Fig 6), supporting the hypothesis that reduction or loss of *adsl-1* expression results in a tyramine deficiency.

**Fig 7:**
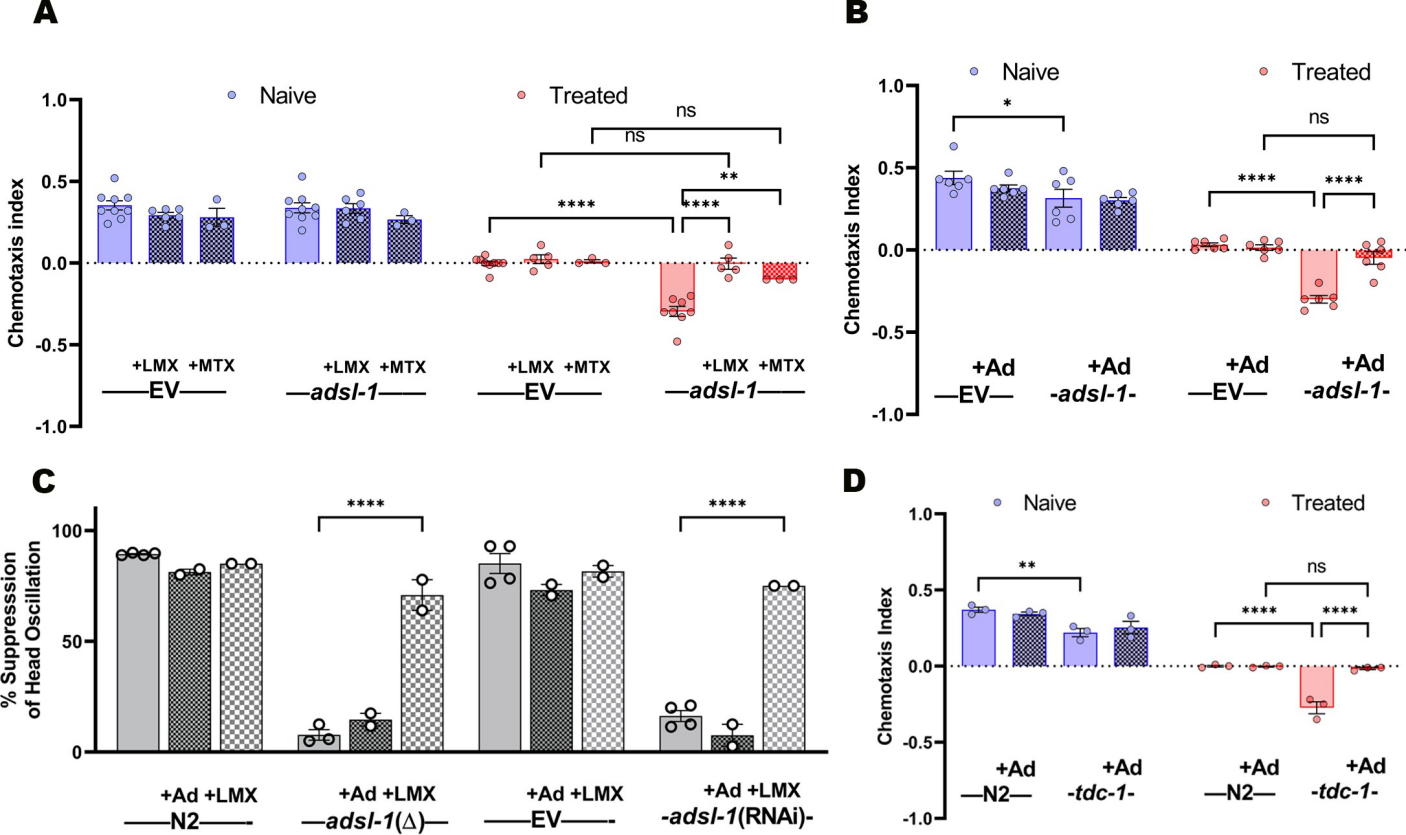
The effect of DNPS inhibitors and adenosine supplementation on *adsl-1*(RNAi) phenotypes. **(A)** Gustatory plasticity assay of empty vector (EV) control and *adsl-1*(RNAi) animals supplemented with 26 nM LMX and 7.5 mM MTX from the L1 stage. *adsl-1*(RNAi) animals treated with each drug had a response comparable to control animals. **(B)** Gustatory plasticity assay of empty vector (EV) control and *adsl-1*(RNAi) animals treated with 10 mM adenosine from the L1 stage. *adsl-1*(RNAi) animals treated with adenosine had a response comparable to the control. Chemotaxis of *adsl-1*(RNAi) animals was slightly depressed relative to the control in this set of experiments. **(C)** Head oscillation assay of control, *adsl-1*(RNAi) and *adsl-1*(tm3328) animals treated with 26 nM LMX or 10 mM adenosine from the L1 stage. Only LMX treatment alleviates the *adsl-1* phenotype. **(D)** Gustatory plasticity assay of N2 control and *tdc-1* mutants treated with 10 mM adenosine from the L1 stage. *tdc-1*(RNAi) animals treated with adenosine had a response comparable to the control. Hatched bars in all panels represent samples with a supplement. Each dot represents the value from a single biological replicate that included (A) 20<n<500, (B) 52<n<205, (C) 19<n<41, and (D) n>200 animals per condition per replicate. Error bars represent standard error of the mean (SEM). * p< 0.05, ** p<0.01, **** p<0.0001 using ANOVA with Bonferroni correction.

We have identified a neurobehavioral phenotype in the *adsl-1*(RNAi) model that appears to result from a tyramine deficiency that is also observed in the loss-of-function *adsl-1(tm2228)* mutant. However, the link between perturbation of purine biosynthesis and production of tyramine is not obvious. In order to investigate potential roles for purine homeostasis or toxic buildup of intermediary metabolites in the tyramine deficiency, we supplemented cultures with adenosine and treated animals with drugs to inhibit DNPS upstream of *adsl-1* function. We treated animals with either the antifolate methotrexate (MTX) or with lometrexol (LMX), an inhibitor of the GART enzyme that acts at multiple steps upstream of ADSL-1 in DNPS. Both treatments designed to inhibit DNPS upstream of ADSL-1 activity restore normal gustatory plasticity to *adsl-1(RNAi)* animals (Fig 7A). Surprisingly, because we expected only one or the other of these treatments to have an effect, adenosine also restored normal gustatory plasticity function to *adsl-1* (Fig 7B).

To determine if the mechanism underlying the effects of DNPS inhibitors and/ or adenosine involved restoring tyramine function, we also examined the effects of LMX and adenosine on head oscillation. While LMX has a robust effect on the head oscillation phenotype of both reduction and loss-of-function *adsl-*1 animals, adenosine does not affect the head oscillation phenotype (Fig 7C). This suggests that the mechanism by which LMX restores gustatory plasticity is by preventing the depletion of tyramine. In contrast, we hypothesized that adenosine might be more directly involved in gustatory plasticity downstream of the effect of tyramine. We tested this hypothesis by examining the effect of adenosine on the gustatory plasticity of tyramine-deficient *tdc-1* mutants. Indeed, adenosine has a positive effect on the gustatory plasticity outcome of *tdc-1* mutants (Fig 7D), suggesting a direct role in behavioral regulation as opposed to a metabolic role in restoring tyramine levels or activity.

## Discussion

*adsl-1* reduction-of-function mutants display an altered response in a salt-aversive gustatory plasticity assay. The altered behavioral response to cue pairing of salt and starvation upon reduction of *adsl-1* function is not due to the animals’ inability to learn. Instead, the animals with reduced *adsl-1* function have an exaggerated avoidance response relative to the indifferent post-training response of control animals. Behavioral responses of the N2 control strain in salt-based associative learning assays vary according to specific training and assay parameters, including training times and liquid versus plate-based training experiences, and avoidance responses in salt-based associative learning assays in *C*. *elegans* have been reported (e.g., [29]). However, to our knowledge there are no other reports of mutants that have an exaggerated (more negative CI) response relative to the control N2 strain as seen in the *adsl-1* reduction-of-function animals and the *tdc-1* and *tyra-2* mutants. This phenotype is striking as a learning difference, with aspects of an exaggerated response, in contrast to a lack of learning.

### Tyramine acts as a neuromodulator in gustatory plasticity

The biogenic amine, tyramine, is both a neuromodulator and neurotransmitter in *C*. *elegans* and has been established as a regulator of behavior in worms [27,32] and in insects [33–40]. The *adsl-1*-associated learning difference appears to be the result of a failure to produce tyramine. Tyramine has been implicated but not directly demonstrated to regulate gustatory plasticity in *C*. *elegans*. Kano *et al*. demonstrated that *tdc-1* mutants have an altered gustatory plasticity response using a four-hour conditioning paradigm followed by an assay for memory retention over a two-hour timespan. They found that *tdc-1* mutants returned to a naïve-like behavior more quickly than controls, indicating an inability to retain memory of the cue pairing experience [29]. While Kano *et al*. did not distinguish between a role for tyramine and octopamine, Rahmani and Chew have suggested that behavioral changes during salt aversion assays are mediated by TDC-1-dependent tyramine synthesis [16]. Our studies support the hypothesis that tyramine is relevant for producing normal responses in gustatory plasticity.

Because tyramine reverts the gustatory plasticity phenotype of *adsl-1*(RNAi) animals, we suspected that tyramine is not produced when *adsl-1* is absent. To support our model, we also sought to examine animals with reduced *adsl-1* function for other tyramine-dependent phenotypes. Production of tyramine is required for a number of behaviors, including suppression of spontaneous reversals, suppression of head oscillations when backing, and another plastic behavior, olfactory aversion to pathogenic bacteria [27,31]. We have previously shown that *adsl-1(tm3328)* animals have an increased rate of spontaneous reversals [12], and we show here that *adsl-1*(RNAi) animals fail to suppress head oscillations when backing. Assessment of olfactory aversion to pathogenic bacteria requires training during the first larval stage. Because sterility and embryonic lethality prevent us from obtaining any *adsl-1*(RNAi)-affected L1 animals and prevent us from collecting sufficient quantities of *adsl-1(tm3328)* animals [9,12], we were unable to assess *adsl-1* reduction or loss-of-function animals in the olfactory aversion assay. However, because the assay for head oscillation involves assessing single animals, we extended the behavioral studies into the loss-of-function *adsl-1(tm3328)* model, confirming that *adsl-1*(RNAi) and *adsl-1(tm3328)* animals share the lack of suppression of head oscillation phenotype with *tdc-1* mutants. Thus, our data support the model that *adsl-1* are deficient in tyramine production.

Currently, there are four known tyramine receptors in *C*. *elegans*. SER-2, TYRA-2, and TYRA-3 are metabotropic receptors, and LGC-55 is the only known ionotropic tyramine receptor [24,28,32,40,41]. TYRA-2 is required for normal gustatory plasticity in our assay. Neither SER-2 nor TYRA-3 is required, but our results do not rule out a role for LGC-55 on its own or for redundancy between receptors. The requirement for TYRA-2 suggests that tyramine acts through a neurohormonal-like mechanism on the metabotropic TYRA-2 receptor. However, a synaptic function via LGC-55 is still possible. LGC-55, TYRA-2, and SER-2 are important in imprint aversion to pathogenic bacteria [31], and LGC-55 mediates tyramine function in head oscillation [31,32]. The tyramine required for regulation of gustatory plasticity is likely produced by the RIM neurons, as they are one of a limited number of *C*. *elegans* cell types that produce tyramine and are also a key component of the neural circuit controlling reversals during locomotion [27]. Thus, tyramine is important for a variety of behaviors in *C*. *elegans*, and it acts through distinct sets of receptors in these functions.

### SAICAR toxicity contributes to tyramine deficiency

Treatments designed to prevent the production of the ADSL substrate SAICAR revert the tyramine-dependent *adsl-1* phenotypes we examined. Our results are consistent with the hypothesis that SAICAR, or possibly SAICAr, toxicity leads to a tyramine deficiency. Both metabolites accumulate in *C*. *elegans* when *adsl-1* is depleted [9,12]. Some experimenters have attributed toxicity to SAICAr by performing SAICAr supplementation [10]. However, Marsac et al demonstrated that supplementation with AICAr to *C*. *elegans* results in accumulation of AICAR, SAICAR and SAICAr [9], and dephosphorylation of SAICAR to form SAICAr has been proposed to be a detoxification mechanism to reduce the toxic accumulation of SAICAR [42]. Thus, the toxic form of the metabolite is unclear as is the mechanism by which SAICAR/r impacts tyramine production or availability. An increase in tyrosine in *adsl-1*(RNAi) animals relative to control animals provided an impetus for us to investigate the role of dopamine and tyramine in *adsl-1*. However, we consider it unlikely that an inability to make tyramine alone would lead to the observed increase in tyrosine levels because so few cells in *C*. *elegans* synthesize tyramine. SAICAR/r may act to specifically prevent tyramine production or may act more generally in disturbing tyrosine metabolism, perhaps influencing levels or activities of other biogenic amines as well. Given that ADSL-related deficits in learning behavior and locomotion in *C*. *elegans* [12], ciliogenesis in human cells and zebrafish, and neurogenesis in zebrafish have been attributed to SAICAR/r toxicity [10,12], we consider it likely that SAICAR/r may have multiple targets.

Surprisingly, supplementation with adenosine also rescued the gustatory plasticity phenotype of *adsl-1*(RNAi) animals but had no effect on the head oscillation suppression phenotype. This result suggests that adenosine does not restore tyramine availability but may play a more direct role in gustatory plasticity, downstream of tyramine function. Evidence for adenosine as a neuromodulator in *C*. *elegans* is emerging [43,44], and our study adds to the growing list of behaviors and physiology effected by adenosine in *C*. *elegans*. But it remains to be seen if the sole adenosine receptor in *C*. *elegans*, ADOR-1, mediates this activity.

### A model for neurobehavioral phenotypes associated with adenylosuccinate lyase deficiency

The availability of model systems to probe metabolic disease and disorder-related symptoms is a huge asset for shedding light on disease etiology [45]. We have extended the *C*. *elegans* model of adenylosuccinate lyase deficiency to include analysis of neurobehavioral phenotypes, which are a largely mysterious aspect of purine metabolic disorders [3]. Our studies have revealed a connection between accumulation of the purine SAICAR and perturbation of tyramine availability, impacting learning behavior in *C*. *elegans*.

How might this work lend insight into links between purine metabolism and behavior in humans? In vertebrates, biogenic amines such as dopamine and serotonin function prominently as neurotransmitters, hormones and neuromodulators with widespread impacts on behavior. Related families of receptors for dopamine and serotonin are found in both vertebrates and invertebrates, including *C*. *elegans*, and again, these signaling molecules regulate behaviors in invertebrates, suggesting that the use of some biogenic amines in control of behavior is likely ancient [46–48]. Other biogenic amines have been labeled trace amines because they are found at significantly lower abundance in the mammalian nervous system. Trace amines may modulate neural function but do not meet the definition of a neurotransmitter. However, trace amines like tyramine and octopamine are found at relatively higher abundance in invertebrates where they do function as neurotransmitters, hormones and neuromodulators, playing a more prominent role in neural signaling than in humans, for example. Unlike the case with the conserved dopamine receptors, the *C*. *elegans* tyramine and octopamine receptors are distinct from the lineage of the trace amine receptors in humans, suggesting that mechanisms to use these signaling molecules evolved independently along the vertebrate and vertebrate lineages. Essentially, tyramine is a neurotransmitter in worms and not in humans. Yet tyramine is a signaling molecule that clearly impacts behavior in worms [49], and tyramine is gaining attention for complex roles in behavior regulation via its TAAR1 receptor in humans as well [46]. It would not be surprising to find an evolutionarily conserved metabolic link between SAICAR/r and trace amine biosynthesis or degradation. Given the interrelatedness of biogenic amines and their synthesis it is possible that reduced ADSL activity exerts effects in ASLD through similar mechanisms affecting availability of these key behavioral modulators.

## Materials and methods

### *C*. *elegans* culture and strains

Strains were maintained on OP50 *Escherichia coli* under standard conditions at 20° C [50]. The N2, MT15620 *cat-2(n4547)*, MT14680 *lgc-55(n4331)*, OH313 *ser-2(pk1357)*, VC125 *tyra-3(ok325)*, MT13113 *tdc-1(n3419)* and MT9455 *tbh-1(n3247)* strains were obtained from the Caenorhabditis Genetics Center (CGC). SSR199 *tyra-2(tm1846) was* obtained from Dr. Supriya Srinivasan’s lab. For all experiments, animals were synchronized as L1 larvae using hypochlorite solution. All experiments were conducted on Day 1 adults unless otherwise noted. HV854 *adsl-1(tm3328/hT2)* is an outcrossed strain. The allele is homozygous sterile and is balanced with hT2 that causes pharyngeal expression of GFP. Non-GFP homozygous *adsl-1(tm3328)* animals were picked for phenotypic analysis.

### RNAi

N2 gravid adults were synchronized as L1 larvae using hypochlorite solution. Animals were then plated onto either HT115 carrying the empty vector RNAi feeding vector (EV) L4440 or *adsl-1(RNAi)* (Source BioScience, Nottingham, UK) and experiments were conducted on Day 1 adults. RNAi knockdown consistently reduces expression by 50–70% [12]. *adsl-1(RNAi)* produces phenotypes such as protruding vulva (Pvl) and sterility, which were used as visible ways to monitor the efficacy of the RNAi; experiments were conducted only on populations with evident protruding vulva (Pvl) and sterile phenotypes.

### LC-MS

LC-MS data was collected according to previous methods [12] performed at the Penn State Metabolomics Core Facility. N2 worms were on EV(RNAi) and *adsl-1(RNAi)* were analyzed. Chlorpropamide normalization factors were first calculated by averaging the raw peak areas of chlorpropamide across EV*(RNAi)* and *adsl-1(RNAi)* samples and then dividing this average by each sample’s chlorpropamide peak area. The raw peak areas of the metabolites for each sample were then normalized using each sample’s respective chlorpropamide normalization factor. The peak area for chlorpropamide was then removed from each sample. Then, total ion chromatogram (TIC) was determined by adding up the normalized peak areas for each respective sample. A TIC normalization factor was determined by averaging the TICs across all samples and then dividing this average by each sample’s TIC. The peak area data was again normalized, this time using the TIC normalization factor. Principle component analysis and identification of potentially significant discriminating features were performed using MetaboAnalyst (v4.0).

### Gustatory plasticity assay

We used the associative learning assay previously described by Saeki and Hukema with some modifications [18,19]. Synchronized L1 larvae were placed on appropriate plates and allowed to mature to Day 1 adults. Knockdown was verified by observable phenotypes (sluggish and protruding vulva) prior to use of animals in the gustatory plasticity assay.

### Starvation-cue pairing

Animals were washed off plates with either CTX buffer or CTX buffer with 100 mM NaCl or KCl, as appropriate, into 15 ml conical tubes. They were allowed to settle by gravity and washed 2 times with the appropriate CTX buffer before being incubated on a rocker for 15 minutes in 1 ml of CTX buffer, with or without salt and food, as appropriate. CTX buffer containing food was prepared from a culture of HT115 grown at 37°C for 15 hours and then diluted to OD_600_ = 0.5 with CTX buffer ± 100 mM NaCl. After the 15-minute incubation, animals were allowed to settle by gravity and used in a chemotaxis assay.

### Experimental plates

1.7% agar in aqueous solution plates were made the week of the experiment and refrigerated before use. The day before the experiment, the plates were removed from the refrigerator and allowed to reach ambient temperature. The plates were divided into four quadrants by marking the bottom of the plate and a 0.7 cm diameter circle was placed in the center. 2 cm from the center of the plate within each quadrant, 8 μl of either 1M sodium azide or 1M sodium azide + 100 mM NaCl were added at the designated “A–attractant’ or ‘C- control’ spots 10 minutes before the animals were to be plated from starvation-cue pairing.

### Chemotaxis assay

Animals were placed in the 0.7 cm diameter circle. Excess CTX buffer (±100 mM salt) was removed with a Kimwipe. Animals were allowed to roam the plate for 30 minutes. Then the number of animals in each quadrant were counted. Animals that did not move from the center were not counted. We then calculated the Chemotaxis Index (CI) as follows:

Chemotaxis index = (# animals in attractant quadrants ‐ # animals in control quadrants) / animals in all quadrants.

### Supplementation experiments

We prepared filter-sterilized stock solutions and added them to OP50-seeded NGM plates to the indicated final concentrations. Following supplementation, we incubated plates at room temperature for 1–2 days before use. Tyramine hydrochloride (Millipore Sigma): 1 M stock solution in water, used at 10 mM plate concentration. Adenosine (Sigma): 117 mM stock solution in water with 10% 1 M NaOH, used at 10 mM plate concentration. Disodium lometrexol (Medkoo Biosciences, Morrisville, NC): Prepared 0.14 mM solution in water, serial diluted to 1.367 μM stock solution, used at 26.3 mM plate concentration. Methotrexate disodium (Fisher Scientific): 1M stock solution in water, used at 7.5 mM plate concentration.

### Head oscillation assay

Gravid adults were placed onto a fresh NGM plate, allowed to lay eggs for 6 hours and then removed. When animals were Day 1 adults, they were transferred onto an empty NGM plate. They were allowed to acclimate for one minute. After 1 minute had elapsed, the animal was stroked along the worm’s body as described [27] and scored according to whether they oscillated their head during the elicited reversal.

### Osmotic ring test

Osmotic avoidance assay was done as previously described with some modifications [51]. Briefly, a 2-cm ring of 4M sodium chloride solution with bromophenol blue was added to a 6-cm unseeded NGM plate and allowed to dry for 5 minutes. Animals were placed in the center of the ring for 10 minutes. After 10 minutes had elapsed, 10 animals were scored in each of three biological replicates according to whether they had crossed the ring. Animals paralyzed on the ring are counted as if they crossed.

### Statistical analysis

We used GraphPad Prism 9 for all statistical analysis. Details for statistical testing are in Figure Legends and provided in S1 Data Set.

## Supporting information

S1 Fig No sensory mechanosensory defects are detected in *adsl-1(RNAi)* animals. Mechanosensation was examined in *adsl-1(RNAi)* and EV control animals by scoring response to **(A)** gentle nose touch and **(B)** posterior body touch. An L4 larva was placed on an empty NGM plate and allowed to acclimate for one minute. After 1 minute had elapsed, the animal was (A) allowed to contact a hair placed in its path (gentle nose touch) or (B) stroked with a hair (posterior body touch). Each of 10 animals was touched ten times, with ten second intervals between each touch. Positive responses were recorded.
(TIF)

S1 Data Set Data and statistical analysis details underlying all graphs. (XLSX)
