# Supplementary figures and images for: Adenylosuccinate lyase deficiency affects neurobehavior via perturbations to tyramine signaling in Caenorhabditis elegans
Source: PLoS Genet. 2023 Sep 29;19(9):e1010974. doi: 10.1371/journal.pgen.1010974 (PMC10566684; doi:10.1371/journal.pgen.1010974)

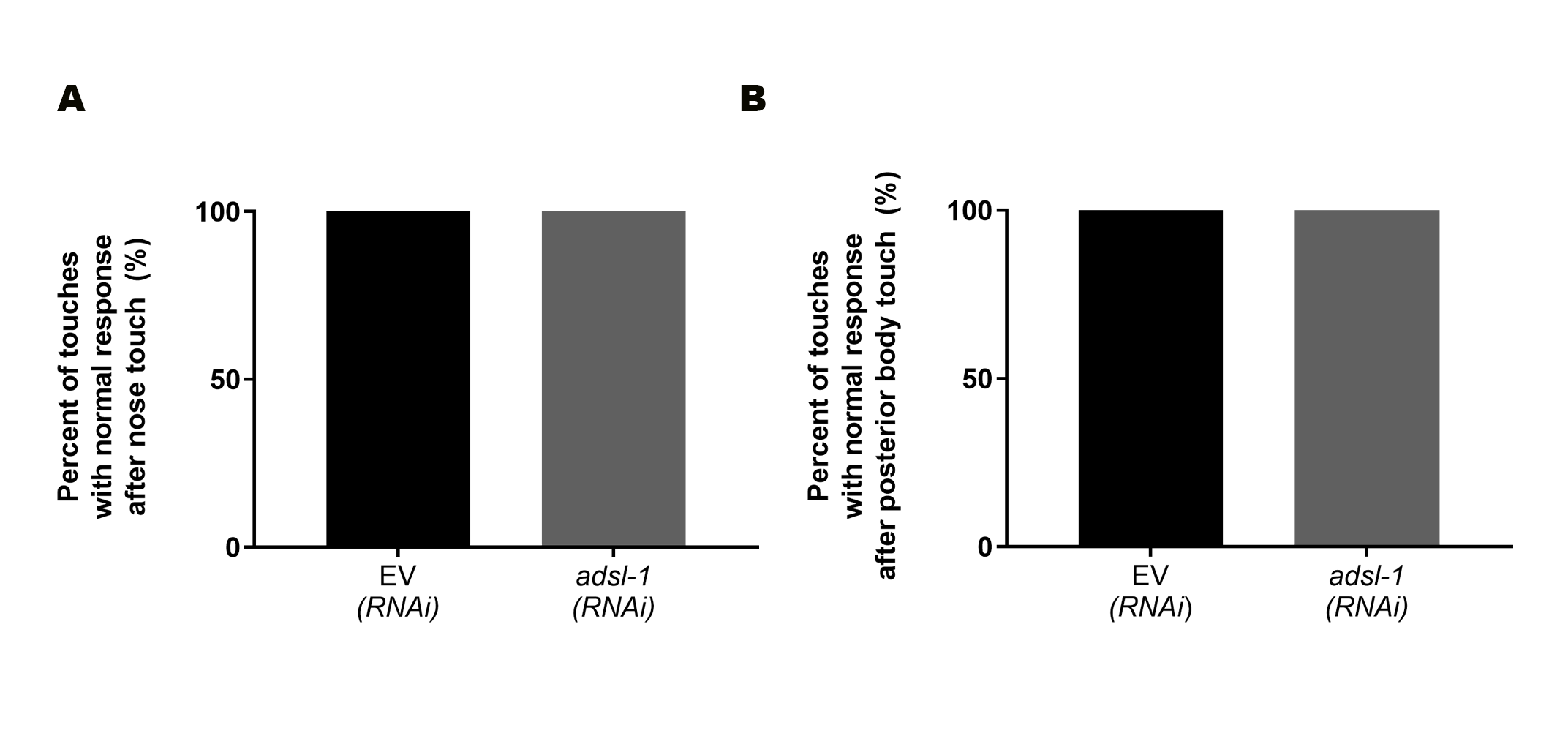

Supplement: S1 Fig — Mechanosensation was examined in adsl-1(RNAi) and EV control animals by scoring response to (A) gentle nose touch and (B) posterior body touch. An L4 larva was placed on an empty NGM plate and allowed to acclimate for one minute. After 1 minute had elapsed, the animal was (A) allowed to contact a hair placed in its path (gentle nose touch) or (B) stroked with a hair (posterior body touch). Each of 10 animals was touched ten times, with ten second intervals between each touch. Positive responses were recorded. (TIF) [file pgen.1010974.s001.tif]
